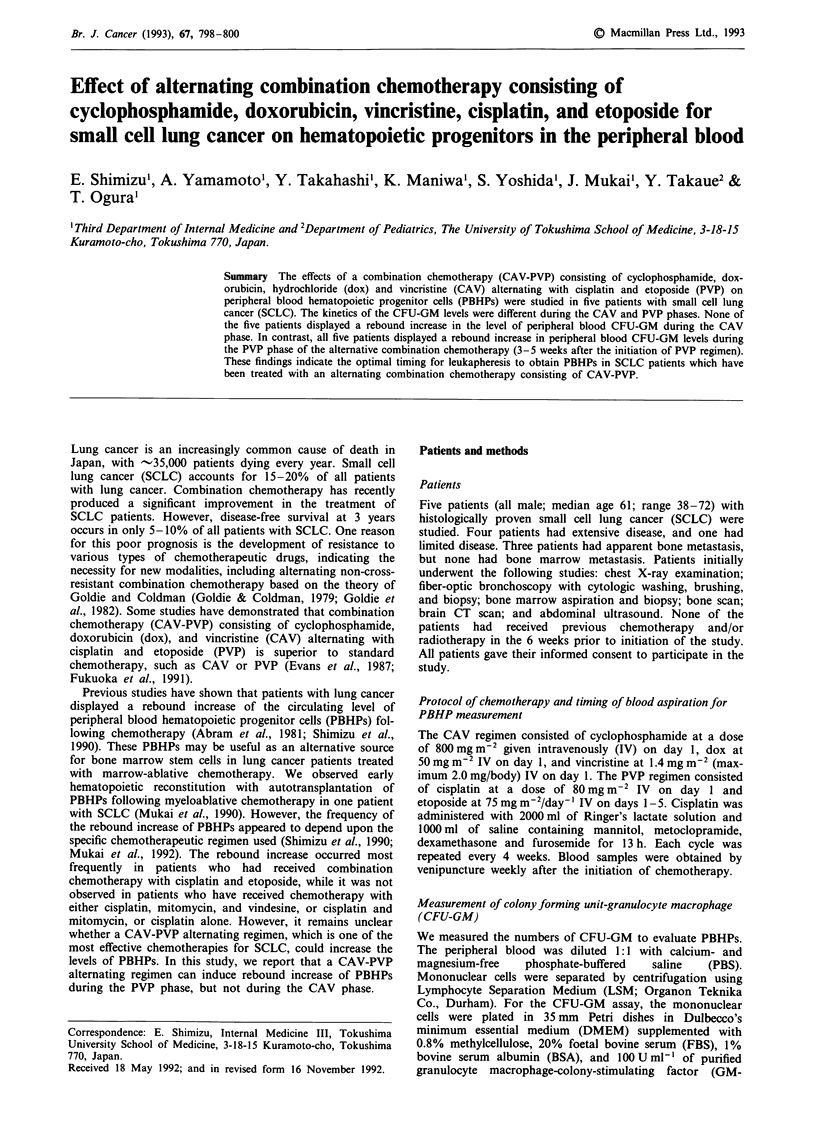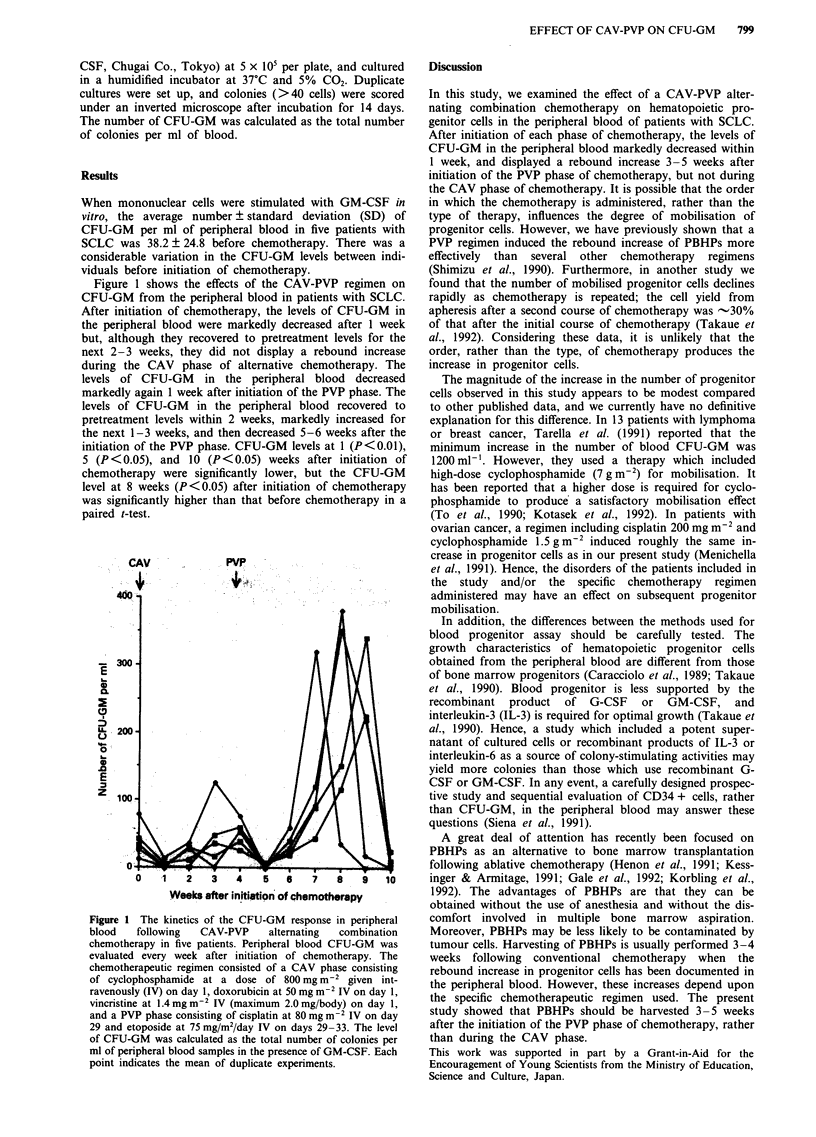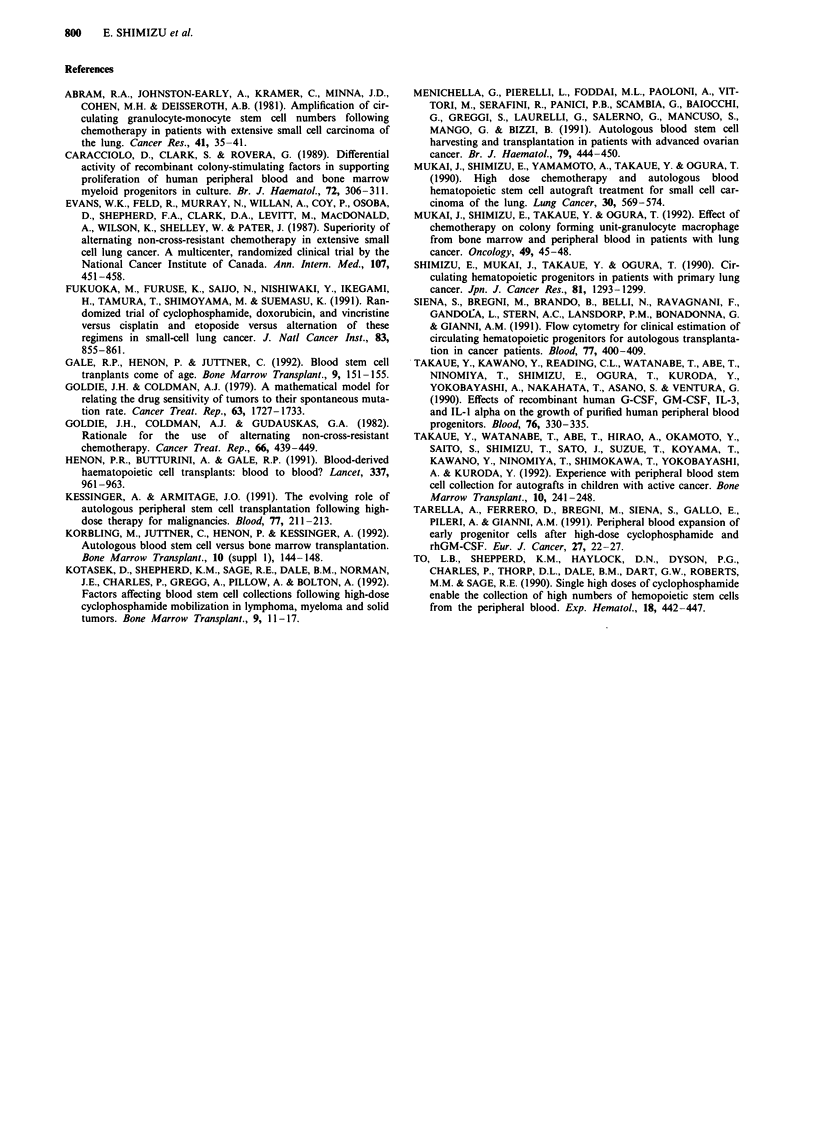# Effect of alternating combination chemotherapy consisting of cyclophosphamide, doxorubicin, vincristine, cisplatin, and etoposide for small cell lung cancer on hematopoietic progenitors in the peripheral blood.

**DOI:** 10.1038/bjc.1993.145

**Published:** 1993-04

**Authors:** E. Shimizu, A. Yamamoto, Y. Takahashi, K. Maniwa, S. Yoshida, J. Mukai, Y. Takaue, T. Ogura

**Affiliations:** Third Department of Internal Medicine, University of Tokushima School of Medicine, Japan.

## Abstract

The effects of a combination chemotherapy (CAV-PVP) consisting of cyclophosphamide, doxorubicin, hydrochloride (dox) and vincristine (CAV) alternating with cisplatin and etoposide (PVP) on peripheral blood hematopoietic progenitor cells (PBHPs) were studied in five patients with small cell lung cancer (SCLC). The kinetics of the CFU-GM levels were different during the CAV and PVP phases. None of the five patients displayed a rebound increase in the level of peripheral blood CFU-GM during the CAV phase. In contrast, all five patients displayed a rebound increase in peripheral blood CFU-GM levels during the PVP phase of the alternative combination chemotherapy (3-5 weeks after the initiation of PVP regimen). These findings indicate the optimal timing for leukapheresis to obtain PBHPs in SCLC patients which have been treated with an alternating combination chemotherapy consisting of CAV-PVP.


Br. J. Cancer (1993), 67, 798-800 © Macmillan Press Ltd., 1993

### Effect of alternating combination chemotherapy consisting of cyclophosphamide, doxorubicin, vincristine, cisplatin, and etoposide for small cell lung cancer on hematopoietic progenitors in the peripheral blood

E. Shimizu[1], A. Yamamoto[1], Y. Takahashi[1], K. Maniwa[1], S. Yoshida[1], J. Mukai[1], Y. Takaue[2] & T. Ogura[1]

[1]*Third Department of Internal Medicine and* [2]*Department of Pediatrics, The University of Tokushima School of Medicine, 3-18-15 Kuramoto-cho, Tokushima 770, Japan.*

**Summary** The effects of a combination chemotherapy (CAV-PVP) consisting of cyclophosphamide, doxorubicin, hydrochloride (dox) and vincristine (CAV) alternating with cisplatin and etoposide (PVP) on peripheral blood hematopoietic progenitor cells (PBHPs) were studied in five patients with small cell lung cancer (SCLC). The kinetics of the CFU-GM levels were different during the CAV and PVP phases. None of the five patients displayed a rebound increase in the level of peripheral blood CFU-GM during the CAV phase. In contrast, all five patients displayed a rebound increase in peripheral blood CFU-GM levels during the PVP phase of the alternative combination chemotherapy (3–5 weeks after the initiation of PVP regimen). These findings indicate the optimal timing for leukapheresis to obtain PBHPs in SCLC patients which have been treated with an alternating combination chemotherapy consisting of CAV-PVP.

Lung cancer is an increasingly common cause of death in Japan, with ~35,000 patients dying every year. Small cell lung cancer (SCLC) accounts for 15–20% of all patients with lung cancer. Combination chemotherapy has recently produced a significant improvement in the treatment of SCLC patients. However, disease-free survival at 3 years occurs in only 5–10% of all patients with SCLC. One reason for this poor prognosis is the development of resistance to various types of chemotherapeutic drugs, indicating the necessity for new modalities, including alternating non-cross-resistant combination chemotherapy based on the theory of Goldie and Coldman (Goldie & Coldman, 1979; Goldie *et al.*, 1982). Some studies have demonstrated that combination chemotherapy (CAV-PVP) consisting of cyclophosphamide, doxorubicin (dox), and vincristine (CAV) alternating with cisplatin and etoposide (PVP) is superior to standard chemotherapy, such as CAV or PVP (Evans *et al.*, 1987; Fukuoka *et al.*, 1991).

Previous studies have shown that patients with lung cancer displayed a rebound increase of the circulating level of peripheral blood hematopoietic progenitor cells (PBHPs) following chemotherapy (Abram *et al.*, 1981; Shimizu *et al.*, 1990). These PBHPs may be useful as an alternative source for bone marrow stem cells in lung cancer patients treated with marrow-ablative chemotherapy. We observed early hematopoietic reconstitution with autotransplantation of PBHPs following myeloablative chemotherapy in one patient with SCLC (Mukai *et al.*, 1990). However, the frequency of the rebound increase of PBHPs appeared to depend upon the specific chemotherapeutic regimen used (Shimizu *et al.*, 1990; Mukai *et al.*, 1992). The rebound increase occurred most frequently in patients who had received combination chemotherapy with cisplatin and etoposide, while it was not observed in patients who have received chemotherapy with either cisplatin, mitomycin, and vindesine, or cisplatin and mitomycin, or cisplatin alone. However, it remains unclear whether a CAV-PVP alternating regimen, which is one of the most effective chemotherapies for SCLC, could increase the levels of PBHPs. In this study, we report that a CAV-PVP alternating regimen can induce rebound increase of PBHPs during the PVP phase, but not during the CAV phase.

Correspondence: E. Shimizu, Internal Medicine III, Tokushima University School of Medicine, 3-18-15 Kuramoto-cho, Tokushima 770, Japan.
Received 18 May 1992; and in revised form 16 November 1992.

### Patients and methods

### *Patients*

Five patients (all male; median age 61; range 38–72) with histologically proven small cell lung cancer (SCLC) were studied. Four patients had extensive disease, and one had limited disease. Three patients had apparent bone metastasis, but none had bone marrow metastasis. Patients initially underwent the following studies: chest X-ray examination; fiber-optic bronchoscopy with cytologic washing, brushing, and biopsy; bone marrow aspiration and biopsy; bone scan; brain CT scan; and abdominal ultrasound. None of the patients had received previous chemotherapy and/or radiotherapy in the 6 weeks prior to initiation of the study. All patients gave their informed consent to participate in the study.

### *Protocol of chemotherapy and timing of blood aspiration for PBHP measurement*

The CAV regimen consisted of cyclophosphamide at a dose of 800 mg $m^{-2}$ given intravenously (IV) on day 1, dox at 50 mg $m^{-2}$ IV on day 1, and vincristine at 1.4 mg $m^{-2}$ (maximum 2.0 mg/body) IV on day 1. The PVP regimen consisted of cisplatin at a dose of 80 mg $m^{-2}$ IV on day 1 and etoposide at 75 mg $m^{-2}$/$day^{-1}$ IV on days 1–5. Cisplatin was administered with 2000 ml of Ringer's lactate solution and 1000 ml of saline containing mannitol, metoclopramide, dexamethasone and furosemide for 13 h. Each cycle was repeated every 4 weeks. Blood samples were obtained by venipuncture weekly after the initiation of chemotherapy.

### *Measurement of colony forming unit-granulocyte macrophage (CFU-GM)*

We measured the numbers of CFU-GM to evaluate PBHPs. The peripheral blood was diluted 1:1 with calcium- and magnesium-free phosphate-buffered saline (PBS). Mononuclear cells were separated by centrifugation using Lymphocyte Separation Medium (LSM; Organon Teknika Co., Durham). For the CFU-GM assay, the mononuclear cells were plated in 35 mm Petri dishes in Dulbecco's minimum essential medium (DMEM) supplemented with 0.8% methylcellulose, 20% foetal bovine serum (FBS), 1% bovine serum albumin (BSA), and 100 U $ml^{-1}$ of purified granulocyte macrophage-colony-stimulating factor (GM-

CSF, Chugai Co., Tokyo) at $5 \times 10^5$ per plate, and cultured in a humidified incubator at 37°C and 5% $CO_2$. Duplicate cultures were set up, and colonies (>40 cells) were scored under an inverted microscope after incubation for 14 days. The number of CFU-GM was calculated as the total number of colonies per ml of blood.

### Results

When mononuclear cells were stimulated with GM-CSF *in vitro*, the average number ± standard deviation (SD) of CFU-GM per ml of peripheral blood in five patients with SCLC was 38.2 ± 24.8 before chemotherapy. There was a considerable variation in the CFU-GM levels between individuals before initiation of chemotherapy.

Figure 1 shows the effects of the CAV-PVP regimen on CFU-GM from the peripheral blood in patients with SCLC. After initiation of chemotherapy, the levels of CFU-GM in the peripheral blood were markedly decreased after 1 week but, although they recovered to pretreatment levels for the next 2–3 weeks, they did not display a rebound increase during the CAV phase of alternative chemotherapy. The levels of CFU-GM in the peripheral blood decreased markedly again 1 week after initiation of the PVP phase. The levels of CFU-GM in the peripheral blood recovered to pretreatment levels within 2 weeks, markedly increased for the next 1–3 weeks, and then decreased 5–6 weeks after the initiation of the PVP phase. CFU-GM levels at 1 ($P<0.01$), 5 ($P<0.05$), and 10 ($P<0.05$) weeks after initiation of chemotherapy were significantly lower, but the CFU-GM level at 8 weeks ($P<0.05$) after initiation of chemotherapy was significantly higher than that before chemotherapy in a paired *t*-test.

**Figure 1** The kinetics of the CFU-GM response in peripheral blood following CAV-PVP alternating combination chemotherapy in five patients. Peripheral blood CFU-GM was evaluated every week after initiation of chemotherapy. The chemotherapeutic regimen consisted of a CAV phase consisting of cyclophosphamide at a dose of 800 mg m$^{-2}$ given intravenously (IV) on day 1, doxorubicin at 50 mg m$^{-2}$ IV on day 1, vincristine at 1.4 mg m$^{-2}$ IV (maximum 2.0 mg/body) on day 1, and a PVP phase consisting of cisplatin at 80 mg m$^{-2}$ IV on day 29 and etoposide at 75 mg/m$^2$/day IV on days 29–33. The level of CFU-GM was calculated as the total number of colonies per ml of peripheral blood samples in the presence of GM-CSF. Each point indicates the mean of duplicate experiments.

### Discussion

In this study, we examined the effect of a CAV-PVP alternating combination chemotherapy on hematopoietic progenitor cells in the peripheral blood of patients with SCLC. After initiation of each phase of chemotherapy, the levels of CFU-GM in the peripheral blood markedly decreased within 1 week, and displayed a rebound increase 3–5 weeks after initiation of the PVP phase of chemotherapy, but not during the CAV phase of chemotherapy. It is possible that the order in which the chemotherapy is administered, rather than the type of therapy, influences the degree of mobilisation of progenitor cells. However, we have previously shown that a PVP regimen induced the rebound increase of PBHPs more effectively than several other chemotherapy regimens (Shimizu *et al.*, 1990). Furthermore, in another study we found that the number of mobilised progenitor cells declines rapidly as chemotherapy is repeated; the cell yield from apheresis after a second course of chemotherapy was ~30% of that after the initial course of chemotherapy (Takaue *et al.*, 1992). Considering these data, it is unlikely that the order, rather than the type, of chemotherapy produces the increase in progenitor cells.

The magnitude of the increase in the number of progenitor cells observed in this study appears to be modest compared to other published data, and we currently have no definitive explanation for this difference. In 13 patients with lymphoma or breast cancer, Tarella *et al.* (1991) reported that the minimum increase in the number of blood CFU-GM was 1200 ml$^{-1}$. However, they used a therapy which included high-dose cyclophosphamide (7 g m$^{-2}$) for mobilisation. It has been reported that a higher dose is required for cyclophosphamide to produce a satisfactory mobilisation effect (To *et al.*, 1990; Kotasek *et al.*, 1992). In patients with ovarian cancer, a regimen including cisplatin 200 mg m$^{-2}$ and cyclophosphamide 1.5 g m$^{-2}$ induced roughly the same increase in progenitor cells as in our present study (Menichella *et al.*, 1991). Hence, the disorders of the patients included in the study and/or the specific chemotherapy regimen administered may have an effect on subsequent progenitor mobilisation.

In addition, the differences between the methods used for blood progenitor assay should be carefully tested. The growth characteristics of hematopoietic progenitor cells obtained from the peripheral blood are different from those of bone marrow progenitors (Caracciolo *et al.*, 1989; Takaue *et al.*, 1990). Blood progenitor is less supported by the recombinant product of G-CSF or GM-CSF, and interleukin-3 (IL-3) is required for optimal growth (Takaue *et al.*, 1990). Hence, a study which included a potent supernatant of cultured cells or recombinant products of IL-3 or interleukin-6 as a source of colony-stimulating activities may yield more colonies than those which use recombinant G-CSF or GM-CSF. In any event, a carefully designed prospective study and sequential evaluation of CD34 + cells, rather than CFU-GM, in the peripheral blood may answer these questions (Siena *et al.*, 1991).

A great deal of attention has recently been focused on PBHPs as an alternative to bone marrow transplantation following ablative chemotherapy (Henon *et al.*, 1991; Kessinger & Armitage, 1991; Gale *et al.*, 1992; Korbling *et al.*, 1992). The advantages of PBHPs are that they can be obtained without the use of anesthesia and without the discomfort involved in multiple bone marrow aspiration. Moreover, PBHPs may be less likely to be contaminated by tumour cells. Harvesting of PBHPs is usually performed 3–4 weeks following conventional chemotherapy when the rebound increase in progenitor cells has been documented in the peripheral blood. However, these increases depend upon the specific chemotherapeutic regimen used. The present study showed that PBHPs should be harvested 3–5 weeks after the initiation of the PVP phase of chemotherapy, rather than during the CAV phase.

This work was supported in part by a Grant-in-Aid for the Encouragement of Young Scientists from the Ministry of Education, Science and Culture, Japan.

### References

ABRAM, R.A., JOHNSTON-EARLY, A., KRAMER, C., MINNA, J.D., COHEN, M.H. & DEISSEROTH, A.B. (1981). Amplification of circulating granulocyte-monocyte stem cell numbers following chemotherapy in patients with extensive small cell carcinoma of the lung. *Cancer Res.*, **41**, 35–41.

CARACCIOLO, D., CLARK, S. & ROVERA, G. (1989). Differential activity of recombinant colony-stimulating factors in supporting proliferation of human peripheral blood and bone marrow myeloid progenitors in culture. *Br. J. Haematol.*, **72**, 306–311.

EVANS, W.K., FELD, R., MURRAY, N., WILLAN, A., COY, P., OSOBA, D., SHEPHERD, F.A., CLARK, D.A., LEVITT, M., MACDONALD, A., WILSON, K., SHELLEY, W. & PATER, J. (1987). Superiority of alternating non-cross-resistant chemotherapy in extensive small cell lung cancer. A multicenter, randomized clinical trial by the National Cancer Institute of Canada. *Ann. Intern. Med.*, **107**, 451–458.

FUKUOKA, M., FURUSE, K., SAIJO, N., NISHIWAKI, Y., IKEGAMI, H., TAMURA, T., SHIMOYAMA, M. & SUEMASU, K. (1991). Randomized trial of cyclophosphamide, doxorubicin, and vincristine versus cisplatin and etoposide versus alternation of these regimens in small-cell lung cancer. *J. Natl Cancer Inst.*, **83**, 855–861.

GALE, R.P., HENON, P. & JUTTNER, C. (1992). Blood stem cell tranplants come of age. *Bone Marrow Transplant.*, **9**, 151–155.

GOLDIE, J.H. & COLDMAN, A.J. (1979). A mathematical model for relating the drug sensitivity of tumors to their spontaneous mutation rate. *Cancer Treat. Rep.*, **63**, 1727–1733.

GOLDIE, J.H., COLDMAN, A.J. & GUDAUSKAS, G.A. (1982). Rationale for the use of alternating non-cross-resistant chemotherapy. *Cancer Treat. Rep.*, **66**, 439–449.

HENON, P.R., BUTTURINI, A. & GALE, R.P. (1991). Blood-derived haematopoietic cell transplants: blood to blood? *Lancet*, **337**, 961–963.

KESSINGER, A. & ARMITAGE, J.O. (1991). The evolving role of autologous peripheral stem cell transplantation following high-dose therapy for malignancies. *Blood*, **77**, 211–213.

KORBLING, M., JUTTNER, C., HENON, P. & KESSINGER, A. (1992). Autologous blood stem cell versus bone marrow transplantation. *Bone Marrow Transplant.*, **10** (suppl 1), 144–148.

KOTASEK, D., SHEPHERD, K.M., SAGE, R.E., DALE, B.M., NORMAN, J.E., CHARLES, P., GREGG, A., PILLOW, A. & BOLTON, A. (1992). Factors affecting blood stem cell collections following high-dose cyclophosphamide mobilization in lymphoma, myeloma and solid tumors. *Bone Marrow Transplant.*, **9**, 11–17.

MENICHELLA, G., PIERELLI, L., FODDAI, M.L., PAOLONI, A., VITTORI, M., SERAFINI, R., PANICI, P.B., SCAMBIA, G., BAIOCCHI, G., GREGGI, S., LAURELLI, G., SALERNO, G., MANCUSO, S., MANGO, G. & BIZZI, B. (1991). Autologous blood stem cell harvesting and transplantation in patients with advanced ovarian cancer. *Br. J. Haematol.*, **79**, 444–450.

MUKAI, J., SHIMIZU, E., YAMAMOTO, A., TAKAUE, Y. & OGURA, T. (1990). High dose chemotherapy and autologous blood hematopoietic stem cell autograft treatment for small cell carcinoma of the lung. *Lung Cancer*, **30**, 569–574.

MUKAI, J., SHIMIZU, E., TAKAUE, Y. & OGURA, T. (1992). Effect of chemotherapy on colony forming unit-granulocyte macrophage from bone marrow and peripheral blood in patients with lung cancer. *Oncology*, **49**, 45–48.

SHIMIZU, E., MUKAI, J., TAKAUE, Y. & OGURA, T. (1990). Circulating hematopoietic progenitors in patients with primary lung cancer. *Jpn. J. Cancer Res.*, **81**, 1293–1299.

SIENA, S., BREGNI, M., BRANDO, B., BELLI, N., RAVAGNANI, F., GANDOLA, L., STERN, A.C., LANSDORP, P.M., BONADONNA, G. & GIANNI, A.M. (1991). Flow cytometry for clinical estimation of circulating hematopoietic progenitors for autologous transplantation in cancer patients. *Blood*, **77**, 400–409.

TAKAUE, Y., KAWANO, Y., READING, C.L., WATANABE, T., ABE, T., NINOMIYA, T., SHIMIZU, E., OGURA, T., KURODA, Y., YOKOBAYASHI, A., NAKAHATA, T., ASANO, S. & VENTURA, G. (1990). Effects of recombinant human G-CSF, GM-CSF, IL-3, and IL-1 alpha on the growth of purified human peripheral blood progenitors. *Blood*, **76**, 330–335.

TAKAUE, Y., WATANABE, T., ABE, T., HIRAO, A., OKAMOTO, Y., SAITO, S., SHIMIZU, T., SATO, J., SUZUE, T., KOYAMA, T., KAWANO, Y., NINOMIYA, T., SHIMOKAWA, T., YOKOBAYASHI, A. & KURODA, Y. (1992). Experience with peripheral blood stem cell collection for autografts in children with active cancer. *Bone Marrow Transplant.*, **10**, 241–248.

TARELLA, A., FERRERO, D., BREGNI, M., SIENA, S., GALLO, E., PILERI, A. & GIANNI, A.M. (1991). Peripheral blood expansion of early progenitor cells after high-dose cyclophosphamide and rhGM-CSF. *Eur. J. Cancer*, **27**, 22–27.

TO, L.B., SHEPPERD, K.M., HAYLOCK, D.N., DYSON, P.G., CHARLES, P., THORP, D.L., DALE, B.M., DART, G.W., ROBERTS, M.M. & SAGE, R.E. (1990). Single high doses of cyclophosphamide enable the collection of high numbers of hemopoietic stem cells from the peripheral blood. *Exp. Hematol.*, **18**, 442–447.